# Extensive MHC class IIβ diversity across multiple loci in the small-spotted catshark (*Scyliorhinus canicula*)

**DOI:** 10.1038/s41598-023-30876-6

**Published:** 2023-03-07

**Authors:** Arnaud Gaigher, Alessia Rota, Fabiana Neves, Antonio Muñoz-Mérida, Javier Blasco-Aróstegui, Tereza Almeida, Ana Veríssimo

**Affiliations:** 1 CIBIO‐InBIO, Research Center in Biodiversity and Genetic Resources, University of Porto, 4485-661 Vairão, Portugal; 2 BIOPOLIS Program in Genomics, Biodiversity and Land Planning, CIBIO, Campus de Vairão, 4485-661 Vairão, Portugal; 3 Research Group for Evolutionary Immunogenomics, Max Planck Institute for Evolutionary Biology, Plön, Germany; 4 Research Unit for Evolutionary Immunogenomics, Department of Biology, University of Hamburg, Hamburg, Germany; 5 Department of Earth and Environmental Sciences, University of Milano-Bicocca, Milan, Italy; 6 Faculty of Sciences, University of Lisbon, Campo Grande 016, 1749-016 Lisbon, Portugal

**Keywords:** Evolution, Immunogenetics, Immunology, MHC class II, Next-generation sequencing

## Abstract

The major histocompatibility complex (MHC) is a multigene family responsible for pathogen detection, and initiation of adaptive immune responses. Duplication, natural selection, recombination, and their resulting high functional genetic diversity spread across several duplicated loci are the main hallmarks of the MHC. Although these features were described in several jawed vertebrate lineages, a detailed MHC IIβ characterization at the population level is still lacking for chondrichthyans (chimaeras, rays and sharks), i.e. the most basal lineage to possess an MHC-based adaptive immune system. We used the small-spotted catshark (*Scyliorhinus canicula*, Carcharhiniformes) as a case-study species to characterize MHC IIβ diversity using complementary molecular tools, including publicly available genome and transcriptome datasets, and a newly developed high-throughput Illumina sequencing protocol. We identified three MHC IIβ loci within the same genomic region, all of which are expressed in different tissues. Genetic screening of the exon 2 in 41 individuals of *S. canicula* from a single population revealed high levels of sequence diversity, evidence for positive selection, and footprints of recombination. Moreover, the results also suggest the presence of copy number variation in MHC IIβ genes. Thus, the small-spotted catshark exhibits characteristics of functional MHC IIβ genes typically observed in other jawed vertebrates.

## Introduction

Natural selection, gene duplication and loss, and high rates of recombination represent the main hallmarks shaping the evolution of multigene families^1,2^. These evolutionary mechanisms often translate into high functional allelic diversity spread across multiple loci and are known to be involved in the evolutionary dynamics of the major histocompatibility complex (MHC) system^3,4^, a multigene family with a crucial role in pathogen resistance in jawed vertebrates^5–7^. Classical MHC class I and class II genes encode for cell-surface glycoproteins directly involved in the recognition of pathogen-derived antigens needed to trigger an adaptive immune response^8,9^.

Due to their essential immunological function, MHC genes have evolved the highest level of genetic diversity known in vertebrates to cope with constantly evolving pathogens during host–pathogen coevolution^4^. For instance, specific MHC genes (human leukocyte antigen, HLA) in human populations can exhibit hundreds to thousands of different alleles^10^. Particularly, the diversity in MHC molecules is not randomly distributed but rather located at amino acid residues directly in contact with the peptide antigen within the peptide-binding domain (PBD; domains α1 and α2 for MHC I, and domains α1 and β1 for MHC II). Such diversity within the PBD should allow for the recognition of a wide variety of pathogen-derived antigens. Therefore, the so-called “pathogen-mediated selection” is considered the main driver of MHC diversity observed in species and natural populations^4,7^.

MHC diversity reflects not only the number of different alleles and the high level of amino acid divergence between alleles, but also the number of duplicated MHC genes. Indeed, MHC evolution is driven also by gene duplication^2,11^, and usually species exhibit multiple MHC genes. However, the number and genomic organization of MHC genes can vary greatly among taxa, and such variability has been observed within the main jawed vertebrate lineages including ray-finned fish, amphibians, reptiles, birds and mammals^12–14^. For instance, the reduced and compact organization of the chicken MHC genes^15^, contrasts with the occurrence of many functional MHC genes and pseudogenes in Passeriformes^16–18^. In addition, the high rates of recombination and gene conversion between duplicated genes, that usually characterize the MHC system, can contribute to shape and maintain the diversity at MHC genes^19–21^. Consequently, providing a reliable and detailed molecular characterization of the MHC region constitute a prerequisite to understand the significance of the MHC diversity in relation to pathogen resistance and individual fitness in natural populations.

Such complex and dynamic evolution of the MHC system, i.e. the multi-copy nature of genes with high levels of allelic diversity and divergence, poses considerable practical challenges in our ability to capture the entire MHC diversity^22–25^. However, recent developments in ultra-deep sequencing provided by Illumina’s technology has revolutionized our access to MHC diversity in terms of costs and accuracy^26,27^. In parallel, the scientific community involved in MHC-related topics has focused in overcoming technical difficulties by providing (i) laboratory protocols to reduce PCR artefacts or to help isolate the complete MHC gene family^23,28^, and (ii) bioinformatic pipelines to process large sequencing datasets and distinguish true alleles from artefacts during MHC genotyping^25,27,29–31^. Still, the laboratory and sequencing strategies needed for screening the MHC system remain species-dependent and are particularly laborious in groups of vertebrates for which limited or no genetic reference sequence data are available, such as chondrichthyans (sharks, rays and chimaeras).

All previously mentioned MHC hallmarks have been extensively characterized in the main jawed vertebrate lineages but remain poorly studied in chondrichthyans. Chondrichthyans are the oldest jawed vertebrates with an MHC-based adaptive immune system^32^ and consequently represent a key group to infer the ancestral and derived states of vertebrate’s adaptive immunity^33^. Pioneer studies isolating MHC class I and II genes in chondrichthyans date back to the 1990’s and relied on three species, the nurse shark (*Ginglymostoma cirratum*, Orectolobiformes)^34–39^, the banded houndshark (*Triakis scyllium*, Carcharhiniformes)^40,41^, and the spiny dogfish (*Squalus acanthias*, Squaliformes)^42^. Some of those early studies showed to some extent polymorphism and recombination at MHC Iα and IIα genes^37,41,43^. Recent advances in chondrichthyan immunogenetics focusing on a larger taxonomic breadth of species have revealed that the MHC system is much more complex and diverse than traditionally thought. Ma et al.^44^ revealed that MHC played a role in response to infection, with variation in expression of MHC IIβ mRNA against a pathogenic bacteria challenge in the whitespotted bamboo shark (*Chiloscyllium plagiosum,* Orectolobiformes). Additionally, multiple divergent MHC lineages, high polymorphism at classical MHC molecules, and occurrence of copy number variation are some of the latest discoveries in other cartilaginous fish taxa^45–49^.

MHC function in cartilaginous fish is still not thoroughly known although previous studies have shown that protein sequences of classical class I and II genes show the same conserved features seen in mammals regarding three-dimensional structure, polymorphism in the peptide-binding region, or highest gene expression in mucosal tissues (gill, intestine, stomach) and spleen consistent with their relevance in immune surveillance in vertebrates (reviewed in^50^). However, there are still many open questions regarding function of MHC proteins in this ancient group of vertebrates. For instance, regarding peptide loading in classical MHC class II proteins in the absence of the non-classical DM molecules^47^, or the efficient activation of CD4 + T-cells upon binding to antigen-presenting MHC class II proteins since the co-receptor of the T-cell receptor, CD4, has not been formally identified and characterized in cartilaginous fish. Likewise, the extensive diversity of non-classical class I molecules recently uncovered in cartilaginous fish^46,48^ is still missing functional data ascertaining the role played by these proteins, and whether it is immune-related or not.

Despite these results, the limited genetic resources available for cartilaginous fish taxa has hindered further understanding on the molecular evolution and diversity of MHC genes at the population level. Consequently, the level of MHC diversity exhibited within a species or population remains poorly examined in this group, and whether the chondrichthyan MHC evolves via similar evolutionary forces observed in other jawed vertebrate lineages needs further investigation. Furthermore, whether and how this diversity is functionally and ecologically meaningful at the individual- and population- levels remains undocumented in chondrichthyans (but see^44^). However, the last few years have been a turning point with the release of a few high-quality chondrichthyan genomes^51^, thus providing a unique opportunity to start investigating the role of MHC diversity in natural populations.

In the present study we characterized the MHC IIβ diversity in a shark species, the small-spotted catshark *Scyliorhinus canicula* (Linnaeus, 1758). This species is an abundant small coastal benthic shark with a broad geographic distribution from Norway to Senegal and across the Mediterranean Sea^52^. Despite its low commercial value, *S. canicula* is frequently captured as bycatch in several demersal trawl, gillnet and longline fisheries along its distribution range^53^. By combining available genomic and transcriptomic resources for *S. canicula* and our own protocol for high-throughput Illumina sequencing of MHC IIβ genes at the population-level, we aimed to (i) characterize the genomic organisation of the MHC IIβ region; (ii) assess gene expression of MHC IIβ genes; (iii) estimate MHC IIβ polymorphism at the population-level; (iv) identify the evolutionary forces involved in shaping MHC IIβ diversity in a shark population. This study represents the first MHC class IIβ characterization at the population level in chondrichthyans, and consequently brings new insights as to whether this group follows the traditional MHC IIβ diversity and evolution hallmarks observed in other jawed vertebrates.

## Material and methods

### Sampling and DNA extraction

Our study focused on a single population of the small-spotted catshark (*S. canicula*, Carcharhiniformes) from southern Portugal (Portimão, Algarve). Forty-one adult individuals were collected in 2014 from landings of locally operating commercial fishing vessels, and sampled for muscle or fin clips preserved in 96% ethanol. DNA was extracted using the EasySpin Genomic DNA Tissue Kit (Citomed, Lisbon, Portugal) following the manufacturer’s instructions.

### Genomic organisation of MHC IIβ

In order to provide insights into the genomic structure of the MHC IIβ region of *S. canicula*, we used the sScyCan1.1 genome (63 × coverage) assembled at the chromosome level and accessible at NCBI (BioProject PRJEB35945 and assembly accession GCF_902713615.1). Available chromosomes were screened to extract MHC IIβ sequences using the protocol established in Almeida et al.^47^. As all MHC IIβ hits were detected within chromosome 13 (spanning approximatively 163 Mbp), we downloaded this chromosome and used the mapping reference implemented in Geneious to access the precise localisation of each MHC IIβ gene and each exon (i.e. the signal peptide, the peptide-binding domain (β1), the immunoglobulin superfamily domain (β2), the connecting piece, the transmembrane domain and the cytoplasmic tail). The MHC sequence input for the mapping included several shark species (including *S. canicula*) from multiple “omic” databases available on NCBI (i.e. Transcriptome Shotgun Assembly, TSA; Sequence Read Archive, SRA; Whole-Genome Shotgun, WGS) (sequences retrieved from Almeida et al.^47^). Using the same procedure, MHC IIα genes were retrieved in order to reconstruct the genomic organization of MHC class II genes.

**Figure 1:**
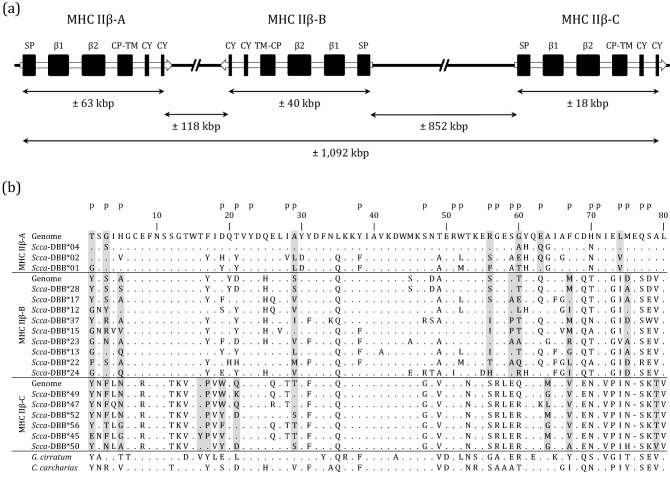
(**a**) Schematic illustration of the genomic organization of MHC class IIβ region using *S. canicula* reference genome sScyCan1.1 (BioProject PRJEB35945). Arrows indicate orientation of the MHC IIβ loci. SP, signal peptide; β1, peptide-binding domain; β2 immunoglobulin superfamily domain; CP, connecting piece; TM, transmembrane domain; CY, cytoplasmic tail. (**b**) Amino acid sequences of MHC IIβ exon 2 alleles (*Scca*-DBB). For readability purpose, only the most divergent alleles are shown. Shaded sites represented sites identified to evolve under positive selection according to the M8 model (CodeML). Amino acid residues expected to interact with antigen peptides are shown above the alignment (p) (Brown et al. 1993). For the purpose of comparison, the nurse shark (*Ginglymostoma cirratum*, AAF82682.1) and white shark (*Carcharodon carcharias* XP_041068366.1) sequences were added.

Analysis of the genome highlighted the occurrence of at least three different MHC IIβ loci, named hereafter as MHC IIβ-A, IIβ-B, and IIβ-C (Fig. 1). The phylogenetic relationships of these genomic sequences with other shark MHC IIβ sequences was visualized via a network using SplitsTree4^54^. Sequence data was extracted from Almeida et al.^47^ and the network was build using MHC IIβ exon 3 (β2 domain) as previously suggested to better infer the evolutionary history among MHC IIβ genes^47^.

### MHC IIβ primer development

We targeted exon 2 of MHC class IIβ genes (β1 domain) that is directly involved in pathogen recognition and is known to exhibit high levels of amino-acid diversity^4^. To develop primers, we took advantage of three different SRA (BioProjects: PRJNA255185, PRJNA504730 PRJNA135005) and one WGS (BioProject: PRJEB35945; WGS Project: CACTIT01) datasets for *S. canicula* freely accessible on NCBI. In addition, WGS data from a closely related species, *Scyliorhinus torazame* (BioProject: PRJDB6260; WGS Project: BFAA01)^55^ was also downloaded from NCBI. These datasets (SRA and WGS) were screened to extract MHC IIβ exon 2 sequences using a protocol as described in Almeida et al.^47^. Finally, we were able to extract additional MHC IIβ sequences from a second *S. canicula* genome dataset kindly provided by Sylvie Mazan (see Acknowledgments). In total, nine and 22 different sequences were retrieved from WGS and SRA datasets, respectively. All the resulting sequences were aligned in Geneious Prime v2.1 using the built-in geneious algorithm. Based on the final alignment of MHC IIβ sequences, two pairs of primers were designed within conserved regions at the junction between the exon 2 and the flanking intronic regions (primers NF2 5’-TCTCACAGGGGCTCACA-3’ with NR2 5’-CCGCTCTCACCTYTCCGG-3’, and primers DF2 5’-CTCTTCTAGGGGCTCATACC-3’ with DR2 5’-CCGCTCTCACCTTTCCTGG-3’) (Supplementary Table S1). The resulting amplicons covered more than 93% of the exon 2 length (238–244 bp). Primers NF2-NR2 were expected to co-amplify two of the MHC IIβ genes present in the *S. canicula* reference genome (sScyCan1.1) (MHC IIβ-A and IIβ-B) while DF2-DR2 was expected to amplify a third gene (MHC IIβ-C).

As an important note, alternative primer combinations were developed (Supplementary Table S1 and Fig. S1) but did not provide the best amplification efficiency (Supplementary Methods); thus, we focused on the dataset generated with the NF2-NR2 and DF2-DR2 primer combinations (as described above) in the current manuscript. Although less effective, the alternative primer combinations yielded data that was used to validate the MHC IIβ sequences detected with NF2-NR2 and DF2-DR2.

### Library preparation and illumina sequencing

Library preparation was performed for Illumina MiSeq sequencing using a two-step PCR amplification procedure. For the first-round of PCR amplification, primers NF2-NR2 and DF2-DR2 were modified by adding 5’-overhangs matching Illumina adapters. All MHC IIβ fragments were amplified on a Bio-Rad T100 Thermal Cycler in a final volume of 10 μl containing 5 μl of 2 × QIAGEN Multiplex PCR Master Mix, 0.4 μl of each primer (NF2-NR2 or DF2-DR2, 10 μM), 3.2 μl of H2O and approximately 1 μl of genomic DNA (10 ng). PCR conditions included an initial denaturation step at 95 °C for 15 min, 30 cycles of denaturation at 95 °C for 45 s, annealing at 58 °C for 30 s (but 60 °C for DF2-DR2) and extension at 72 °C for 30 s. A final step at 60 °C for 10 min was used to complete amplicon extension. The PCR products were cleaned using AMPure XP Beads (0.97x) (Beckman Coulter/Agencourt), 80% ethanol, and buffer EB. Quality of cleaned PCR products was checked on 2% agarose gel.

In the second-round of PCR amplification, unique barcodes and Illumina adaptors were added to each sample. The indexing PCR was carried out in a final volume of 14 µL containing 7 µl of 2 × Kapa HiFi Hot Start, 0.7 µl of each of two indexes (P7 and P5, 10 µM), 2.8 µl of H2O and 2.8 µl of cleaned PCR products. The thermocycler conditions included an initial denaturation step at 95 °C for 3 min, 10 cycles of denaturation at 95 °C for 30 s, annealing at 55 °C for 30 s and extension at 72 °C for 30 s. A final step at 72 °C for 5 min was used to complete extension. The indexed PCR products were cleaned using AMPure XP Beads (0.8x), 80% ethanol, and buffer EB, and then checked on 2% agarose gel. Cleaned PCR products were pooled equimolar into a NF2-NR2 pool and a DF2-DR2 pool. These pools were quantified using Epoch Microplate Spectrophotometer (Agilent) and normalized to 20 nM. Quality and fragment size of each pool were assessed using a 2200 TapeStation (Agilent), followed by validation using the KAPA Library Quantification Kit (KAPA Biosystem, Inc., Wilmington, USA) for Illumina sequencing platforms according to the manufacturer’s protocol. The two validated pools of samples were combined into a single library using a 2:1 ratio in favour of NF2-NR2 products as more alleles should be amplified with this primer pair (from 2 gene copies) compared to DF2-DR2 (single gene). The final library was adjusted to a concentration of 12 pM and sequenced with a 250-bp paired-end Illumina MiSeq v2 kit. A total of 20% PhiX was added to the run to allow enough nucleotide diversity to properly identify clusters on the flow cell. Reliability of the sequencing was evaluated by including six replicated samples from independent PCRs in the same run. Library preparation and Illumina sequencing were performed at the sequencing facility of the Centre for Molecular Analysis (CTM) in CIBIO-InBIO (Vairão, Porto, Portugal).

Three additional Illumina Miseq runs were performed with different primer combinations and/or Illumina protocols, but using identical *S. canicula* samples across runs (Supplementary Table S2). The Illumina outputs for those runs were only used to compare and validate MHC IIβ sequences between replicate samples from different runs.

### Illumina raw data processing and MHC genotyping

Reads generated with the Illumina MiSeq sequencing strategy were demultiplexed using BASESPACE (basespace.illumina.com). Adapter sequences, low-quality reads (-q 30) and reads shorter than 100 bp (-m 100) were removed with Cutadapt^56^. The resulting dataset was processed using DADA2 pipeline^57^ with the following steps: (i) quality filtering (i.e. discard low-quality reads, reads with ambiguous nucleotides and expected errors higher than 2) and primer trimming, (ii) error learning using the DADA2 algorithm, (iii) dereplication of identical reads into unique sequences, (iv) merging paired reads with full identity in the overlap region, and (v) filtering out potential chimeric sequences. Finally, an amplicon sequence variant (ASV) table was created and used for subsequent filtering procedures for MHC genotyping.

Distinguishing true alleles from artefacts during MHC genotyping is a crucial but challenging step to gain reliable genotypes^22,30,58^. Our MHC IIβ genotyping was performed in line with MHC standard approaches for high‐throughput sequencing^25–27,29–31,59^. Across the whole dataset (ASV table), samples with less than 100 sequences per amplicon, and variants with a maximum sequence count per amplicon lower than 10 were discarded. All remaining variants were aligned in Geneious Prime v2.1, and those differing from the targeted loci were removed (confirmed as not being MHC loci based on blastn searches). From this reduced ASV table, the per-amplicon variant frequency (PAVF) was calculated (i.e*.* the frequency of each variant within each amplicon) and variants with frequencies < 1% were automatically considered as artefacts and discarded. FASTA files for each amplicon were created to detect and remove potential low frequency artefact variants such as chimeras, indels or single substitution errors from parental true variants/alleles of higher frequencies. Low frequency variants found in high frequency in other amplicons (considered as true variants/alleles) were considered as artefacts and discarded based on (i) the assumption that variants should generally amplify similarly across amplicons (but see^25^), and (ii) the incongruence in the occurrence of these low frequency variants between replicates.

Based on the different primer combinations and runs, the reliability of our MHC genotyping protocol was evaluated with (i) 74 replicates within runs, (ii) 85 replicates between different runs, and (iii) 75 replicates between primer combinations.

### Gene expression of MHC IIβ loci

Locus-specific gene expression estimation was performed to test if all MHC IIβ loci were expressed, and thus functionally relevant. For this purpose, we used seven SRA datasets from RNAseq projects of *S. canicula* available on NCBI, namely three SRA datasets from BioProject PRJNA255185 (belonging to liver, pancreas and brain tissues), three SRA datasets from BioProject PRJNA504730 (composed by immature ovary, mature testis and immature testis) and an SRA dataset from BioProject PRJNA135005 (from stage 24–30 embryos). Sequence read files from the different tissues were pre-processed using Trimmomatic^60^ to remove adapters and trim low quality bases at the 3' and 5' ends. The resulting high-quality reads per tissue were mapped against a query dataset with the predicted mRNAs for *S. canicula* MHC IIβ genes obtained from the reference genome (sScyCan1.1). Reads mapping against any of the query MHC IIβ sequences were used to perform a de novo assembly through rnaSPAdes software^61^ thus building all the isoforms with any expression in that tissue. Assembled isoforms were used as reference sequences to re-map the tissue reads and get read counts, transcripts per million (TPM) and fragments per kilobase of transcripts per million fragments mapped (FPKM) through the RNA-Seq by Expectation Maximization (RSEM) by calling *rsem-prepare-reference* with specific parameter –bowtie2 and the *rsem-calculate-expression* with default parameters^62^. Given the different BioProjects used here and the inherent variability in sequence coverage and protocols used in RNA extraction and library preparation, no comparisons in expression levels were made among tissues.

### Characterization of MHC IIβ exon 2

All confirmed alleles for *S. canicula* were named according to the standard nomenclature^63^ and deposited in GenBank. Genetic diversity indices were estimated in DnaSP 6^64^ for each MHC IIβ locus, namely the number of polymorphic sites, the average number of nucleotide differences (k) and the average number of pairwise differences per base pair (π), while the amino acid p-distances were obtained in MEGA 11^65^.

To assess the phylogenetic relationships between MHC IIβ exon 2 alleles of *S. canicula* we computed a Neighbor-Net network using SplitsTree4^54^ based on uncorrected p-distances. Such representation is particularly suited to the MHC system which is known to evolve by gene duplication and recombination. In addition, another network was built to visualize the phylogenetic relationships of those alleles with other shark MHC IIβ exon 2 sequences. Sequence data was extracted from our previous work in Almeida et al.^47^.

To infer codon sites evolving under positive selection, we used the maximum likelihood site-models in CodeML implemented in PAML 4^66^. As each of the inferred MHC loci may be under different selective pressures, analyses were performed independently for each locus. The likelihood ratio test of positive selection was carried out comparing models M7 versus M8 (neutral vs proportion of sites under positive selection, respectively). When the best-fit model was M8, sites under positive selection were identified through the Bayes Empirical Bayes (BEB) method. Trees used as input for the selection analysis were obtained with MrBayes 3^67^. Best substitution models identified with MEGA were JC + G for MHC IIβ-A and MHC IIβ-B, and K80 + G for MHC IIβ-C. For each locus, two independent Markov Chain Monte Carlo runs (MCMC) of 5 × 10^6^ generations with a sampling every 1000 generations were performed, with posterior probabilities being calculated after a burn-in of 25%. Convergence was assessed using the average standard deviation of split frequencies between runs, the estimated sample size and the potential scale reduction factor (PSRF) using MrBayes 3^67^ and Tracer^68^. As the signal of selection may be sensitive to the tree topology, the CodeML analysis was repeated five times with five different trees randomly chosen from the posterior distribution of tree topologies (the best tree was always included). In addition, the impact of historical selection on the MHC IIβ exon 2 sequences was determined with the one-tailed Z-test implemented in MEGA 11^65^. These analyses were run for each locus on three data partitions: (i) the entire exon; (ii) codons of the PBS (peptide-binding sites) exclusively; and (iii) codons of the non-PBS exclusively (PBS were inferred from Human HLA, Brown et al. 1993). The average rates of synonymous (dS) and nonsynonymous (dN) substitutions were computed for each locus and partitions using the Nei–Gojobori method (with Jukes–Cantor correction) in MEGA 11^65^.

Recombination events and putative recombinant sequences were inferred using multiple methods implemented in different programs. First, we used RDP4 software^69^ to apply six different algorithms, including 3Seq, BootScan, Chimerae, MaxChi, RDP, and SiScan. Analyses were performed using default settings with a highest acceptable *p*-value of 0.05 and Bonferroni correction for multiple comparisons. Second, gene conversion events were tracked using Geneconv 1.81^70^ with 10,000 permutations and a g-scale value of 0 (i.e. mismatches not allowed). Third, we performed the Phi test of recombination (Φw)^71^ implemented in SplitsTree 4^54^, and estimated the minimal number of historical recombination events^72^ using the four-gamete test in DnaSP.

To gauge our sampling effort in allele discovery, we carried out permutation tests to assess the number of alleles detected for a given number of individuals sampled (see^73^). As not all individuals were the same between the two sets of primers used for MHC IIβ exon 2 amplification (NF2-NR2 vs DF2-DR2), the analyses were performed first with individuals of each set of primers (both n = 33) and secondly only considering individuals in common between the two sets of primers (n = 25). For each sample size (n, from 1 to 25 or 33), we randomly extracted n individuals and counted the number of different alleles detected. The procedure was repeated 500 times for each sample size to calculate the mean and the standard deviation.

## Results

### Genomic organisation of three MHC IIβ loci

**Figure 2:**
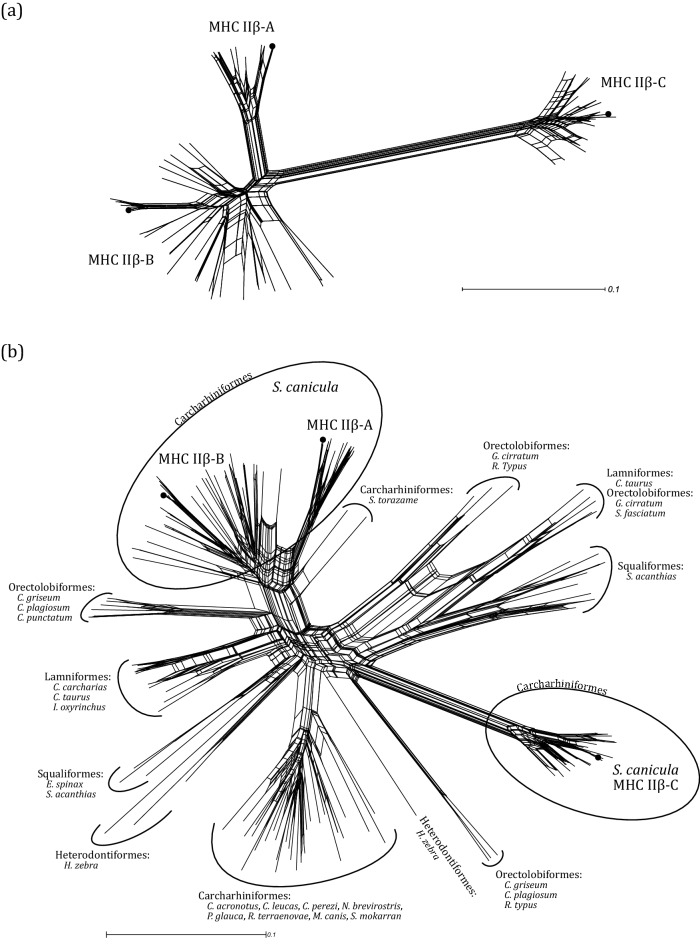
(**a**) Phylogenetic network of small-spotted catshark MHC IIβ exon 2 alleles. (**b**) Phylogenetic network of MHC IIβ exon 2 sequences comparing the small-spotted catshark to other shark species. Sequences were extracted from Almeida et al.^47^. In both networks, the three black dots represent the sequences extracted from the *S. canicula* reference genome sScyCan1.1 (BioProject PRJEB35945).

Using the reference genome sScyCan1.1 assembled at the chromosome level, we were able to reconstruct the genomic organisation of MHC IIβ region. Three different MHC IIβ loci were detected and spread within a range of approximately 1 Mbp on chromosome 13 (163 Mbp) (Fig. 1a). NCBI reference sequences for those three genes were XM_038816327.1, XM_038815839.1, and XR_005462827.1. The expectation of three MHC IIβ loci was in line with the level of genetic divergence between alleles observed in the phylogenetic network (Fig. 2a). Moreover, the phylogenetic relationships of the retrieved alleles with regards to those of other Elasmobranchs suggested that the three MHC IIβ loci of *S. canicula* belong to the DBB lineage (nomenclature *MhcScca-DBB*; Supplementary Fig. S2). On the genome, MHC IIβ-A and MHC IIβ-B loci were physically closer to each other compared to the MHC IIβ-C locus (Fig. 1a). In addition, MHC IIβ-A and MHC IIβ-C loci were oriented in the same direction compared to MHC IIβ-B locus (Fig. 1a). For each loci, the different exons (coding for the signal peptide, the peptide-binding domain (β1), the immunoglobulin superfamily domain (β2), the connecting piece, the transmembrane domain and the cytoplasmic tail) were retrieved and followed the expected organization of vertebrate MHC IIβ genes (Fig. 1a). MHC IIβ-A gene was approximatively two and three times longer in bp than MHC IIβ-B and MHC IIβ-C genes, respectively, suggesting overall large intronic regions. Two out of the three sequences retrieved from the genome were identical to alleles detected in our population samples (Fig. 2a).

The detailed organisation of MHC IIα loci in regards to MHC IIβ is depicted in the Supplementary Information (Supplementary Fig. S3). In total, four different MHC IIα loci were retrieved, all located next to the MHC IIβ loci (NCBI reference sequences for MHC IIα XM_038815840.1, XM_038815841.1, XM_038815843.1, and XM_038815844.1).

### Gene expression of MHC IIβ loci

The gene expression analysis confirmed the presence of transcripts from all MHC IIβ loci identified in *S. canicula*. Transcripts from MHC IIβ-A and IIβ-B were predominant in all tissues surveyed while IIβ-C transcripts were detected only in pancreas, immature ovary, and mature testis (Supplementary Table S3). In general, MHC IIβ-C transcripts showed lower gene expression levels when compared to those from the remaining two loci (Supplementary Table S3).

### Illumina sequencing and validation of MHC IIβ genotyping

Primer combination NF2-NR2 co-amplified the exon 2 of MHC IIβ-A and MHC IIβ-B, while primer combination DF2-DR2 amplified MHC IIβ-C. Based on the 41 samples from the South of Portugal (including replicates), Illumina sequencing yielded 260,173 reads (in average 4518 and 2488 reads per individual for NF2-NR2 and DF2-DR2 primer combinations, respectively). After data cleaning and filtering, we retained 179′441 reads (in average 3041 and 1900 reads per individual for NF2-NR2 and DF2-DR2 primer combinations, respectively). Out of initially 70 and 23 variants for NF2-NR2 and DF2-DR2 primer combinations, respectively, 44 and 20 were considered as true alleles during our genotyping procedure. During these filtering and genotyping steps, eight samples for each primer combination were automatically discarded due to low coverage or unclear genotype profile.

We validated our genotyping procedure based on 234 replicates split as (i) replicates within run, (ii) replicates between different runs, and (iii) replicates of different primer combinations. Ultimately, 507 and 233 different comparisons between replicates were possible for NF2-NR2 and DF2-DR2 amplification respectively, as for instance, eight individuals had nine different Illumina sequencing outputs (i.e. 36 comparisons per individual for NF2-NR2). Overall, 88% and 92% of comparisons showed identical genotypes for NF2-NR2 and DF2-DR2 amplification respectively, highlighting the good repeatability of our sequencing and genotyping protocols. The incongruences between replicates were identified as being due to (i) contaminations, or (ii) different amplification efficiency between primer combinations (e.g. three variants were confirmed as true alleles with the NF2-NR2 primer combination, while the replicates with other primers failed to properly amplify them).

### MHC IIβ exon 2 characterization

**Figure 3:**
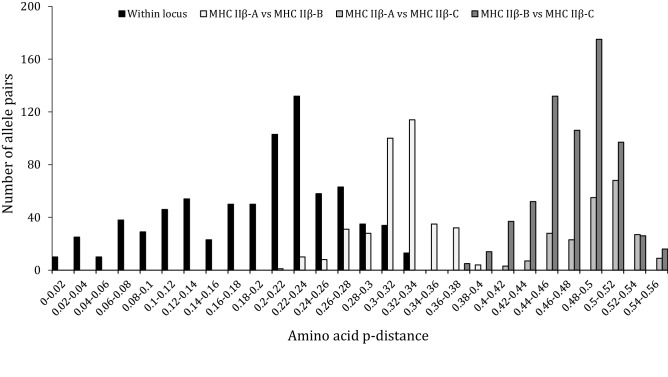
Histogram of the amino acid p-distance between MHC IIβ exon 2 alleles in small-spotted catshark. Black bars represent p-distance between alleles within each MHC IIβ locus, while the three shades of grey bars represent p-distances between MHC IIβ loci.

Based on 41 different individuals, we detected a high level of allelic diversity across the three different loci (i.e. MHC IIβ-A, IIβ-B, and IIβ-C loci) with a total of 64 alleles (*Scca*-DBB*01 to *Scca*-DBB*64, GenBank accession numbers OQ123732-OQ123795) (Table 1). However, the MHC IIβ-B locus had the highest genetic diversity levels, followed by MHC IIβ-C and by MHC IIβ-A locus (lowest diversity levels) (Table 1). All nucleotide alleles translated into unique amino acid alleles. No alleles showed evidence of non-functionality, such as stop codons or frameshift mutations. All alleles from the MHC IIβ-C locus displayed a deletion of three nucleotides at the end of exon 2 compared to the two other loci (Fig. 1b). In line with the phylogenetic network (Fig. 2a), MHC IIβ-C was highly divergent from the MHC IIβ-A and IIβ-B loci (average amino acid p-distance between MHC IIβ-C and IIβ-A, and MHC IIβ-C and IIβ-B, respectively: 0.493 and 0.472) (Fig. 3), while MHC IIβ-A and IIβ-B loci were more similar (average amino acid p-distance: 0.317) (Fig. 3). Most of the divergence between MHC IIβ-A and IIβ-B loci was located at the end of the exon 2 by diagnostic amino acid difference (Fig. 1b). MHC IIβ diversity exhibited in the small-spotted catshark seems unique in respect to other shark sequences as *S. canicula* MHC IIβ exon 2 sequences clustered together (Fig. 2b).

**Table 1:** Genetic diversity at the small-spotted catshark MHC IIβ exon 2 (β1 domain).

	Number of alleles	Number of sites	S	k	π (S.D.)	AA distance (S.E.)
MHC IIβ-A	11	244	37	15.82	0.065 (0.009)	0.140 (0.027)
MHC IIβ-B	33	244	89	30.94	0.127 (0.004)	0.229 (0.030)
MHC IIβ-C	20	238	40	14.94	0.063 (0.004)	0.105 (0.022)
MHC IIβ combined	64	241	135	51.68	0.217 (0.006)	0.339 (0.031)

S, number of polymorphic sites; k, average number of nucleotide differences; π, average number of pairwise differences per base pair; AA distance, amino acid pairwise distance.

Our analysis performed with CodeML showed evidence of positive selection on specific codon sites for the three different MHC IIβ loci. We detected six, nine and 12 sites evolving under positive selection for MHC IIβ-A, IIβ-B, and IIβ-C loci, respectively (Fig. 1b). The region under selection was mostly congruent between loci (five, four and six sites shared between MHC IIβ-A and IIβ-B, MHC IIβ-A and IIβ-C, and MHC IIβ-B and IIβ-C, respectively). Three quarters of sites identified as positively selected were residues supposed to directly interact with antigen peptides (Fig. 1b). In line with these results, the Z-test of positive selection was only significant for PBS data partition in all loci (Table 2). The dN/dS ratio was much higher for PBS than non-PBS residues (Table 2).

**Table 2:** Average rates of nonsynonymous substitutions (dN) and synonymous substitutions (dS) for all sites, PBS and non-PBS in the MHC IIβ exon 2.

	Sites	dN (S.E.)	dS (S.E.)	dN/dS	Z-test	*P*
MHC IIβ-A	All	0.078 (0.018)	0.040 (0.022)	1.950	1.437	0.077
	PBS	0.236 (0.058)	0.049 (0.043)	4.816	2.992	**0.002**
	Non-PBS	0.023 (0.009)	0.037 (0.025)	0.622	− 0.535	1.000
MHC IIβ-B	All	0.141 (0.025)	0.132 (0.035)	1.068	0.260	0.398
	PBS	0.393 (0.089)	0.196 (0.084)	2.005	2.428	**0.008**
	Non-PBS	0.059 (0.012)	0.106 (0.040)	0.556	− 1.236	1.000
MHC IIβ-C	All	0.070 (0.018)	0.052 (0.019)	1.346	0.787	0.216
	PBS	0.159 (0.053)	0.026 (0.014)	6.115	2.837	**0.003**
	Non-PBS	0.036 (0.015)	0.065 (0.029)	0.554	− 1.047	1.000

Standard errors (S.E.) were estimated by bootstrap with 1000 replications. Significant (*P* < 0.05) Z-test for positive selection (HA: dN > dS) are highlighted in bold.

Similarly, based on several recombination methods, we found evidence of both intra- and inter-locus recombination. Intra-locus recombination was detected for each of the three loci, with a total of 15 and 10 recombination events based on the RDP4 and Geneconv analysis, respectively (Supplementary Table S4). Inter-locus recombination was observed mostly between MHC IIβ-A and IIβ-B loci with four and 13 events using RDP4 and Geneconv analysis, respectively, while only once between MHC IIβ-A and IIβ-C loci (in RDP4 analysis only) (Supplementary Table S4).

**Figure 4:**
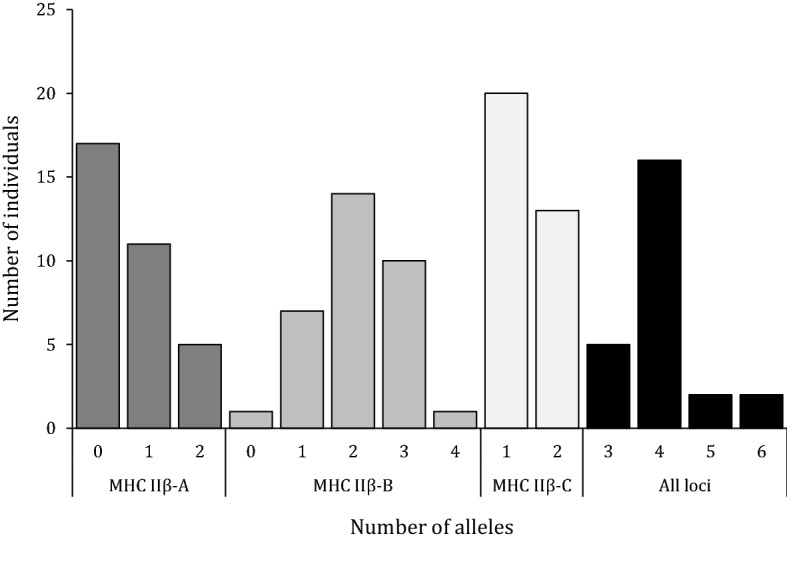
Distribution of number of alleles per individual in the small-spotted catshark. For each locus independently n = 33, but when considering all loci n = 25.

At the individual level, when considering the two primer pair combinations, between three and six different alleles per individual were observed, with the majority of individuals (65%) having four alleles (‘All loci’ in Fig. 4), in agreement with the expectation of the presence of three different loci. With primers NF2-NR2 (MHC IIβ-A and MHC IIβ-B loci), between two and four alleles per individual were amplified. However, and surprisingly, half of individuals lacked alleles supposedly belonging to the MHC IIβ-A locus, and one individual lacked alleles from MHC IIβ-B locus (Fig. 4). In turn, one individual harbored four alleles all supposedly from MHC IIβ-B locus (Fig. 4). For primers DF2 and DR2 (MHC IIβ-C), we observed a higher proportion of individuals with only one allele compared to individuals with two alleles (61% vs 39%, Fig. 4).

**Figure 5:**
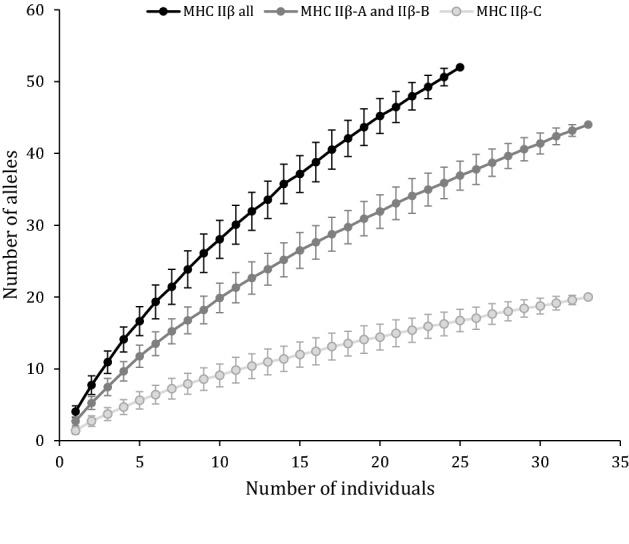
New allele discovery in relation to the number of individuals sampled. The bars indicate the standard deviation of the estimated mean. The black line includes only individuals with complete MHC IIβ genotypes (successful amplification of both sets of primers: NF2-NR2 and DF2-DR2) (n = 25), while dark and light grey lines include only individuals with successful amplification of NF2-NR2 (MHC IIβ-A and -B, n = 33) and DF2-DR2 (MHC IIβ-C, n = 33), respectively.

Plotting the number of alleles detected for a given number of individuals sampled revealed no allele saturation, suggesting that sampling more individuals will still significantly increase the discovery of new alleles in this population (Fig. 5).

## Discussion

In the present study, we performed a detailed characterization of MHC IIβ genes and genetic diversity at the population level for a cartilaginous fish. Using the small-spotted catshark (*Scyliorhinus canicula*, Carcharhiniformes) as a model species, we reconstructed the genomic MHC IIβ region, validated an MHC IIβ genotyping protocol for the amplification of the exon 2 (encoding the PBD), and revealed the typical features of functional MHC genes in basal jawed vertebrates including expression of the three different MHC IIβ loci, a high polymorphism across multiple loci, and footprints of positive selection and recombination.

### Inference of MHC IIβ lineages

High-quality reference genome sequences are prerequisites to accurately reconstruct the genomic architecture of complex gene families with duplicated loci, such as the MHC^74^. Using an available high-quality genome of the small-spotted catshark (sScyCan1.1, BioProject PRJEB35945), we identified three different MHC IIβ loci within the same chromosome (chromosome 13). In line with this, the MHC IIβ allelic diversity at the population level fall into three distinct phylogenetic clusters, each of them including the reference genomic sequences of a single loci. These results were supported with a second available draft genome (see Acknowledgments). In our recent work, two divergent and ancient MHC IIβ lineages (DAB and DBB) were identified in sharks from available genome and transcriptome data^47^, however the three loci detected in the small-spotted catshark cluster only within the DBB lineage (Supplementary Fig. S2). The presence of the DBB lineage appeared widely spread across shark taxa, while DAB lineage was restricted to few species across three different Orders including Squaliformes, Lamniformes, and Orectolobiformes^34,36,39,47^, but not in Carcharhiniformes (to which *S. canicula* belongs). We tested several primers to amplify the exon 2 of MHC IIβ DAB lineage in the small-spotted catshark, however all our attempts failed to isolate sequence data (data not shown). Three reasons could explain this result: (i) the DAB lineage is evolutionary absent in this species or even at a higher taxonomic level (i.e. Carcharhiniformes) and could have been lost during MHC evolution, (ii) the designed primers were too divergent to efficiently amplify the DAB lineage, and (iii) the investigated populations and individuals may lack the DAB lineage due to copy number variation (CNV). This last scenario was previously revealed in the nurse shark (*Ginglymostoma cirratum*, Orectolobiformes) with CNV at MHC IIα^37^ and MHC IIβ genes^47^. Whether the small-spotted catshark or other Carcharhiniformes possess the DAB lineages therefore require a deeper investigation.

Altogether our results suggest the presence of three DBB loci, but not all individuals possessed the different MHC IIβ loci. This is particularly evident in the results obtained with primers NF2-NR2, which co-amplified two different loci and up to four alleles per individual. Indeed, half of the individuals were lacking MHC IIβ-A alleles with this primer combination. Likewise, the majority had only a single MHC IIβ-C allele. Several reasons could explain this pattern. (i) The distinction of loci based on the allelic divergence and phylogenetic analyses may be misleading, i.e. a specific locus can retain highly divergent MHC alleles while highly similar alleles can be shared between loci due to homogenization by gene conversion. This latter mechanism was previously proposed to play a predominant role in shaping diversity at MHC genes^2,20,21,75^. (ii) The designed primers may have caused biased amplification in favour of some alleles (e.g. MHC IIβ-B alleles), while less-amplified alleles could have been discarded due to low coverage during our filtering steps. Such bias is a well-known issue in MHC studies and remains undetected if several primer combinations are not tested^23,25,76^. (iii) MHC IIβ-A and IIβ-C loci may be biologically absent in some individuals due to CNV, a pattern previously described in many other species^26,27,77–79^, including the nurse shark^47^. Scenarios (ii) and (iii) may explain the lack the MHC IIβ-A alleles in some individuals, but also suggest the presence of an additional MHC locus as 11 individuals had more than two MHC IIβ-B alleles.

Our data highlights the main challenges of most MHC studies, i.e. to infer the exact number of loci and to assign alleles to a specific locus. In our case, the scenario (iii) seems the most likely as all primers tested for our candidate gene approach and the different types of datasets analysed (genomes and transcriptomes) converge to similar outcomes, i.e. at least three different *Scca*-DBB loci with potential CNV. Further studies are needed to ascertain which is the most likely scenario in the small-spotted catshark, for instance by sequencing other MHC IIβ regions (e.g. intron 1, exon 3)^80^, generating long-read genomic data to confirm genomic rearrangement and the presence of CNV^12,14^, or by using family data to improve allele scoring and haplotype reconstructions^26^.

### Typical features of functional MHC genes

Studies targeting MHC genes in sharks remain scarce (Supplementary Table S5), which contrast with the extensive number of MHC studies for other jawed vertebrates. Nevertheless, high polymorphism was previously suggested at MHC IIα in the nurse shark^37^ and MHC Iα in the banded houndshark^41^ based on 12 and 22 individuals, respectively. Population-based MHC IIβ studies in a shark species remain absent, or limited to family data^39,47^. The levels of genetic diversity detected in the MHC IIβ genes of the small-spotted catshark and the evolutionary forces shaping this diversity are in line with what was previously observed in other jawed vertebrate populations. We found high levels of allelic diversity and divergence, with 64 functional MHC IIβ alleles discovered from only 41 small-spotted catshark, which seems proportionally higher compared to many other vertebrates (amphibians^81,82^, birds^29,83–85^ and mammals^86–89^), but not compared to specific taxa having particularly high MHC IIβ diversity due to many duplicated loci, such as in Passeriformes^90–93^ or in several fish species^94,95^. In addition, our current data points out that the number of MHC IIβ alleles for this population is probably underestimated and a larger sampling effort would increase the discovery of new alleles. Indeed, more than 100 wild animals are required for sampling completeness of MHC IIβ alleles in several species^73,91,96^. The extensive allelic diversity coupled with the high level of divergence between alleles in the small-spotted catshark MHC IIβ therefore provides the unique potential for individuals to interact with a wide range of pathogen-derived peptides and for the population to adapt to a given parasite community^97,98^.

We detected clear footprints of selection shaping MHC IIβ exon 2 diversity in the small-spotted catshark, regardless of locus. The large majority of polymorphic sites as well as sites evolving under positive selection were found at peptide-binding sites based on Human HLA^99^. This pattern of historical positive selection has been commonly observed in jawed vertebrates, and often proposed to result from selective pressure imposed by pathogens^7^. However, the actual selective agents underpinning MHC IIβ diversity in contemporary small-spotted catshark populations remain unknown, and whether this functional MHC IIβ diversity associate to pathogen resistance or susceptibility needs further testing with larger samples sizes. Indeed, few studies revealed strong associations between MHC diversity and pathogen resistance although this may result from low statistical power owing to small sample sizes and/or the generally small effect sizes of MHC variation on fitness-related traits or immunocompetence^100^. In addition to this, a good understanding of the pathogen community of the target species remains a prior step for future studies testing the association of MHC variation and individual fitness-related traits.

Recombination and gene conversion were also shown to shape MHC IIβ sequence diversity in the small-spotted catshark. These processes were previously suggested to shape MHC allelic diversity in other shark species^35,37,41^. These are significant drivers of MHC diversity by generating new MHC alleles and haplotypes and can create diversity at a faster pace than point mutations^21,101^. In line with the inter-locus recombination events detected in our dataset, the close location of MHC IIβ gene duplicates in the small-spotted catshark genome may favour allele shuffling by gene conversion between duplicates^102^. Although these processes may promote the evolution of high-diversity MHC haplotypes, it may also restrict the co-segregation of co-adapted alleles^29^. Likely, all these evolutionary processes act in concert to generate the MHC diversity in the small-spotted catshark, but pointing out their relative contribution in maintaining the levels of MHC diversity remains challenging^7^.

## Concluding remarks

This study will serve as a stepping stone for future work in Elasmobranch immunogenetics but also opens up opportunities to pursue follow-up studies on host–pathogen dynamics and co-evolution, as well as in ecology and conservation of our target species. For instance, the high MHC diversity revealed in the small-spotted catshark make MHC markers a suitable alternative candidate to the neutral ones in improving the spatial resolution of population structure^103,104^. This feature can improve stock identification and the sustainable long-term management of this commercially exploited shark.

## Supplementary Information

Supplementary Information.

## Data Availability

Sequences of the 64 MHC IIβ alleles are available in GenBank (accession numbers OQ123732-OQ123795). In addition, a FASTA file with the MHC IIβ transcripts from gene expression for all BioProjects is available in the Mendeley Data repository (https://data.mendeley.com/datasets/96njfygf69/1).
