# Supplementary material for: Extensive MHC class IIβ diversity across multiple loci in the small-spotted catshark (Scyliorhinus canicula)
Source: Sci Rep. 2023 Mar 7;13:3837. doi: 10.1038/s41598-023-30876-6 (PMC9992475; doi:10.1038/s41598-023-30876-6)

Supplementary Information for

Extensive MHC class IIβ diversity across multiple loci in the small-spotted catshark (*Scyliorhinus canicula*)

Arnaud Gaigher[1,2,3,4], Alessia Rota[1,2,5], Fabiana Neves[1,2], Antonio Muñoz-Mérida[1,2], Javier Blasco-Aróstegui[1,2,6], Tereza Almeida[1,2] & Ana Veríssimo[1,2]

[1] CIBIO-InBIO, Research Center in Biodiversity and Genetic Resources, University of Porto, 4485-661 Vairão, Portugal

[2] BIOPOLIS Program in Genomics, Biodiversity and Land Planning, CIBIO, Campus de Vairão, 4485-661 Vairão, Portugal

[3] Research Group for Evolutionary Immunogenomics, Max Planck Institute for Evolutionary Biology, Plön, Germany

[4] Research Unit for Evolutionary Immunogenomics, Department of Biology, University of Hamburg, Hamburg, Germany

[5] Department of Earth and Environmental Sciences, University of Milano-Bicocca, Milan, Italy

[6] Faculty of Sciences, University of Lisbon, Campo Grande 016, 1749-016 Lisbon, Portugal

Corresponding author: Arnaud Gaigher

E-mail: arnaud.gaigher@gmail.com

Supplementary Methods

The genome highlights the occurrence of at least three different MHC IIβ loci, named here after as MHC IIβ-A, IIβ-B, and IIβ-C. Based on this, 10 primers were developed corresponding to six different primer combinations, including three to target simultaneously MHC IIβ-A and IIβ-B loci (NF and NR) and three for the MHC IIβ-C locus (DF and DR) (Table S1). NF1-NR1 and DF1-DR1 primer combinations cover the entire exon 2 and a small part of the flanking introns. While NF2-NR2 and DF2-DR2 combinations target almost the full exon 2 sequence (93%), NF3-NR2 and DF3-DR2 combinations only amplify 77% of the exon 2 (Supplementary Figure S1). Preliminary analyses revealed that NF1-NR1 and DF1-DR1 primer combinations yield i) a large number of non-targeted sequences, and ii) different sequence sizes (due to a microsatellite in the upstream region of the exon 2 and of a repetitive sequence downstream the exon 2). NF3-NR2 and DF3-DR2 primer combinations generated sequences lacking a polymorphic region of the exon 2. Therefore, we decided to focus our analyses in the rest of this manuscript on the dataset based on NF2-NR2 and DF2-DR2 primer combinations.

Table S1: Primer combinations used for the amplification of exon 2 of MHC IIβ genes in the small-spotted catshark (*S. canicula*). Primer combinations in bold correspond with the definitive ones used to characterize the MHC diversity in *S. canicula*. The other combinations were used to validate the MHC IIβ diversity detected with NF2-NR2 and DF2-DR2 primer combinations. F refer to Forward and R to reverse.

Amplification	Primer combination	Sequence (5′→3′)	Size (bp)
MHC IIβ-A<br>MHC IIβ-B	NF1<br>NR1	F: GTCTCCCGGCAGTAACTCTG<br>R: CCCAGTTACARGGGAACC	351-418
**MHC IIβ-A**<br>**MHC IIβ-B**	**NF2**<br>**NR2**	**F: TCTCACAGGGGCTCACA**<br>**R: CCGCTCTCACCTYTCCGG**	**244**
MHC IIβ-A<br>MHC IIβ-B	NF3<br>NR2	F: GGATGTGTGTTTAACAGCTCC<br>R: CCGCTCTCACCTTTCCGG	206
MHC IIβ-C	DF1<br>DR1	F: TCCTCATCTGGAGCCTGATC<br>R: CGCACCAGGCAGACTGTGAC	398-447
**MHC IIβ-C**	**DF2**<br>**DR2**	**F: CTCTTCTAGGGGCTCATACC**<br>**R: CCGCTCTCACCTTTCCTGG**	**238**
MHC IIβ-C	DF3<br>DR2	F: GGGTGTCGGTTTAACAGCACC<br>R: CCGCTCTCACCTTTCCTGG	202

Table S2: Description of the different Illumina runs performed in our study. N refer to the number of PCR samples. Run in bold is the definitive one used to characterize the MHC diversity in *S. canicula*.

Run	Primer combination	N	PhiX	Illumina technology
Run 1	NF1-NR1<br>DF1-DR1	58	25%	250-bp paired-end MiSeq Nano kit
Run 2	NF2-NR2<br>DF2-DR2<br>NF3-NR2<br>DF3-DR2	280	15%	150-bp paired-end MiSeq Nano kit
Run 3	NF2-NR2<br>DF2-DR2<br>NF3-NR2	166	5%	250-bp paired-end MiSeq kit
**Run 4**	**NF2-NR2**<br>**DF2-DR2**	**1177**	**20%**	**250-bp paired-end MiSeq kit**

Table S3: Gene expression of MHC IIβ transcripts retrieved from three different BioProjects available on NCBI for *Scyliorhinus canicula*, using RSEM. Gene expression levels are provided as expected read counts (Expected counts), Transcripts Per Kilobase Million (TPM) and Fragments Per Kilobase Million (FPKM), for each BioProject analysed, tissue type (Tissues), MHC IIβ loci, and transcript.

Bioproject	Tissue	Loci	Transcript identification	Length	Eff. length	Exp. count	TPM	FPKM
PRJNA135005	embryos		21AB1_938_rev	938	863	172.0	464487	940117
PRJNA135005	embryos		21AB2_338_rev	338	263	32.0	283563	573929
PRJNA135005	embryos		21AB3_149_rev	149	74	8.0	251950	509944
PRJNA135005	embryos		22AB1_919_cov_6.400227_g0_i0	919	844	148.0	354167	868096
PRJNA135005	embryos		22AB2_353_cov_4.873418_g1_i0	353	278	45.0	326931	801339
PRJNA135005	embryos		22AB3_132_rev	132	57	9.0	318902	781657
PRJNA135005	embryos		23AB1_1032_cov_7.160804_g0_i0	1032	957	110.0	219431	459770
PRJNA135005	embryos		23AB2_730_rev_cov_8.942280_g0_i1	730	655	98.0	285629	598473
PRJNA135005	embryos		23AB3_237_rev_cov_10.180000_g1_i0	237	162	42.0	494939	1037037
PRJNA255185	liver		29AB1_1489_cov_321.941667_g0_i0	1489	1320.49	1866.1	262096	226504
PRJNA255185	liver		29AB2_1489_cov_320.745139_g0_i1	1489	1320.49	2576.4	361863	312723
PRJNA255185	liver		29AB3_1411_rev_cov_343.243025_g0_i2	1411	1242.49	1664.3	248429	214693
PRJNA255185	liver		29AB4_1411_rev_cov_285.428047_g0_i3	1411	1242.49	24.8	3708	3204
PRJNA255185	liver		29AB5_328_rev_cov_28.111111_g1_i0	328	160.84	107.5	123905	107079
PRJNA255185	brain		30AB1_1533_cov_181.426550_g0_i0	1533	1371.81	1465.4	492867	359283
PRJNA255185	brain		30AB2_1533_cov_170.149596_g0_i1	1533	1371.81	763.4	256783	187186
PRJNA255185	brain		30AB3_1533_cov_135.599730_g0_i2	1533	1371.81	744.3	250350	182496
PRJNA255185	pancreas		31AB1_1314_cov_72.397260_g1_i0	1314	1013.08	22.9	35334	36037
PRJNA255185	pancreas		31AB2_1275_rev_cov_93.449251_g1_i1	1275	974.08	514.1	823974	840365
PRJNA255185	pancreas		31AB3_317_cov_2.409836_g2_i0	317	17.82	0.0	0	0
PRJNA255185	pancreas	MHC IIβ-C	31D1_1333_rev_cov_15.515873_g0_i0	1333	1032.08	46.0	69616	71000
PRJNA255185	pancreas	MHC IIβ-C	31D2_1289_rev_cov_16.220395_g0_i1	1289	988.08	45.0	71076	72490

(cont.)

(cont.)

BioProject	Tissue	Loci	Transcript identification	Length	Eff. length	Exp. count	TPM	FPKM
PRJNA504730	immature ovary		89AB1_1465_cov_161.117232_g0_i0	1465	1304.02	1410.0	700043	548311
PRJNA504730	immature ovary	MHC IIβ-C	89D1_1374_cov_48.940377_g1_i0	1374	1213.02	562.0	299957	234942
PRJNA504730	mature testis		90AB1_1470_rev_cov_35.657284_g0_i0	1470	1310.12	121.0	188297	153930
PRJNA504730	mature testis		90AB2_1470_rev_cov_35.632653_g0_i1	1470	1310.12	130.0	202302	165379
PRJNA504730	mature testis		90AB3_1470_rev_cov_35.223786_g0_i2	1470	1310.12	143.0	222532	181917
PRJNA504730	mature testis		90AB4_1470_rev_cov_34.827586_g0_i3	1470	1310.12	205.0	319015	260791
PRJNA504730	mature testis	MHC IIβ-C	90D1_183_rev_cov_0.761194_g1_i0	183	30.05	1.0	67853	55469
PRJNA504730	immature testis		91AB1_1480_cov_152.827393_g0_i0	1480	1327.98	805.0	309602	233867
PRJNA504730	immature testis		91AB2_1474_cov_183.054035_g0_i1	1474	1321.98	926.0	357755	270240
PRJNA504730	immature testis		91AB3_1474_cov_170.413333_g0_i2	1474	1321.98	861.0	332643	251271

Table S4: Recombination events estimated by several methods.

	No. of sequences	Rm	Φw test	3Seq	BootScan	Chimerae	MaxChi	RDP	SiScan	Geneconv
MHC IIβ-A	11	7	$p = 0.039$	1	0	1	1	0	0	1
MHC IIβ-B	33	18	$p < 10^{-3}$	5	0	2	7	0	6	2
MHC IIβ-C	20	9	$p < 10^{-3}$	1	0	1	2	0	2	7
MHC IIβ-AB	44	22	$p = 0.002$	5	0	2	10	0	4	18[a]
MHC IIβ-AC	31	13	$p = 0.161$	2	0	0	2	0	3	20[b]
MHC IIβ-BC	53	18	$p = 0.007$	1	0	1	5	0	5	19[c]
MHC IIβ-ABC	64	19	$p = 0.001$	3	0	2	6	0	6	26[d]

Rm, minimum number of recombination events

[a] 13 global fragments are between alleles from MHC IIβ-A and IIβ-B loci

[b] all global fragments are between alleles from the same locus (20 MHC IIβ-C)

[c] all global fragments are between alleles from the same locus (2 MHC IIβ-B, 17 MHC IIβ-C)

[d] 8 global fragments are between alleles from MHC IIβ-A and IIβ-B loci, while the rests are between alleles from the same locus (2 MHC IIβ-B, 16 MHC IIβ-C)

Table S5: List of studies characterizing MHC class I and II genes in sharks. N represents the number of shark specimen. Reviews are not included.

**Study**	**MHC class**	**Species**	**N**
This study	MHC IIβ	*Scyliorhinus canicula*	41
Almeida *et al.* 2021	MHC I	Multiple species	1 to 6[a]
Okamura *et al.* 2021	MHC W-category	Multiple species	1 to 2[b]
Almeida *et al.* 2020a	MHC I	Multiple species	1 to 6[a]
Almeida *et al.* 2020b	MHC IIβ and IIα	Multiple species	1 to 6[a]
Ma *et al.* 2013	MHC IIβ	*Chiloscyllium plagiosum*	3
Wang *et al.* 2003	MHC I	*Squalus acanthias*	1
Bartl 2001	MHC IIβ	*Ginglymostoma cirratum*	1
Ohta *et al.* 2000	MHC II and I	*Ginglymostoma cirratum*	17 and 39[c]
Okamura *et al.* 1997	MHC I	*Triakis scyllium*	22[d]
Bartl *et al.* 1997	MHC I	*Ginglymostoma cirratum*<br>*Heterodontus francisci*	1<br>1
Bartl & Weissman 1994	MHC IIβ	*Ginglymostoma cirratum*	1
Kasahara *et al.* 1993	MHC IIα	*Ginglymostoma cirratum*	12
Hashimoto *et al.* 1992	MHC I	*Triakis scyllium*	1
Kasahara *et al.* 1992	MHC IIα	*Ginglymostoma cirratum*	1

[a] Sequences extracted from transcriptomic and genomic data available on NCBI, one to six adult specimens per species in BioProject

[b] Sequences obtained from two individuals of *Triakis scyllium* (from Okamura *et al.* 1997) and sequences extracted from database searches on NCBI and Ensembl

[c] 17 and 39 pups from two different families, sequences only obtained from shark family of 17 pups

[d] Only 14 out of 22 individuals are unrelated (eight are from similar families)

Figure S1: Schematic illustration of primer positions for the amplification of MHC IIβ exon 2 in the small-spotted catshark. N refer to primers with the simultaneously amplification of MHC IIβ-A and IIβ-B loci while D to primers for the MHC IIβ-C locus.

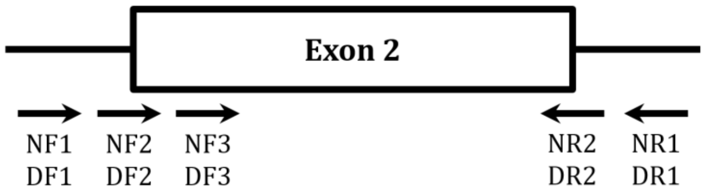

Figure S2: Phylogenetic network of small-spotted catshark sequences in relation to other shark MHC IIβ sequences. The network was build using MHC IIβ exon 3 (β2 domain). Red dots represent the three MHC IIβ exon 3 sequences extracted from the *S. canicula* genome (sScyCan1.1). Black dots are *S. canicula* sequences from other datasets.

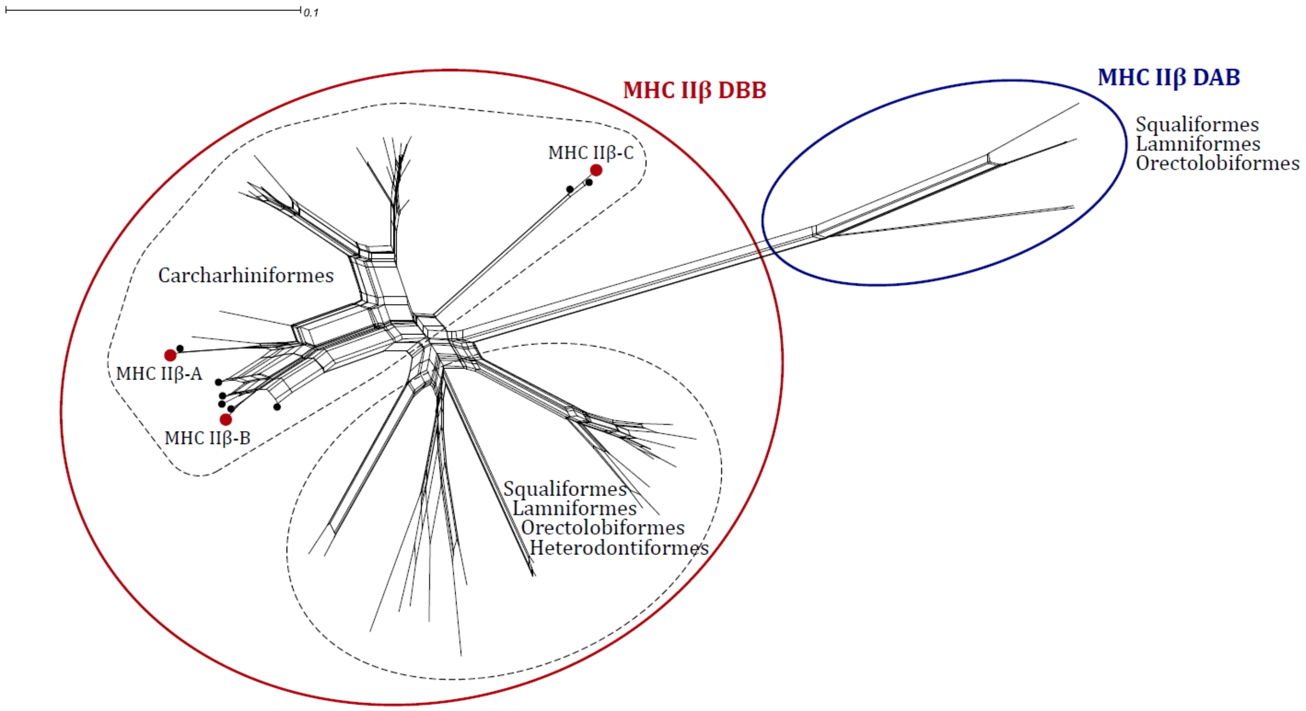

Figure S3: Schematic illustration of the genomic organization of MHC class II region (MHC IIβ and MHC IIα genes) using *S. canicula* reference genome sScyCan1.1 (BioProject PRJEB35945). NCBI reference sequences are for MHC IIα XM_038815840.1, XM_038815841.1, XM_038815843.1, and XM_038815844.1, and for MHC IIβ XM_038816327.1, XM_038815839.1, and XR_005462827.1.

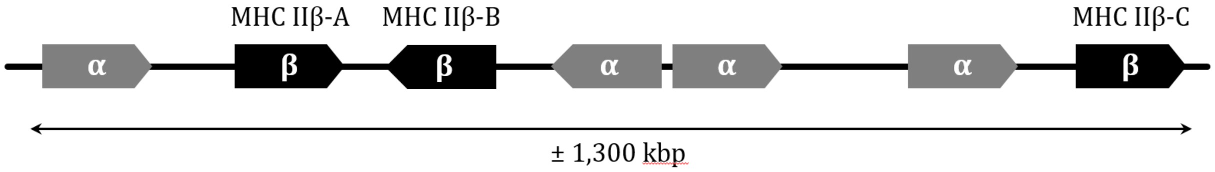

Supplement: Supplementary file 1 — Supplementary Information. [file 41598_2023_30876_MOESM1_ESM.pdf]
